# Individuals Prioritize the Reach Straightness and Hand Jerk of a Shared Avatar over Their Own

**DOI:** 10.1016/j.isci.2020.101732

**Published:** 2020-11-10

**Authors:** Takayoshi Hagiwara, Gowrishankar Ganesh, Maki Sugimoto, Masahiko Inami, Michiteru Kitazaki

**Affiliations:** 1 Department of Computer Science and Engineering, Toyohashi University of Technology, Toyohashi, Aichi, Japan; 2 UM-CNRS Laboratoire d'Informatique de Robotique et de Microelectronique de Montpellier (LIRMM), 161, Rue Ada, Montpellier, France; 3 Department of Information and Computer Science, Keio University, Yokohama, Kanagawa, Japan; 4 Research Center for Advanced Science and Technology, The University of Tokyo, Bunkyo-ku, Tokyo, Japan; 5 Department of Computer Science and Engineering, Toyohashi University of Technology, Toyohashi, Aichi, Japan

**Keywords:** Behavioral Neuroscience, Cognitive Neuroscience

## Abstract

Cyber space enables us to “share” bodies whose movements are a consequence of movements by several individuals. But whether and how our motor behavior is affected during body sharing remains unclear. Here we examined this issue in arm reaching performed by a shared avatar, whose movement was generated by averaging the movements of two participants. We observed that participants exhibited improved reaction times with a shared avatar than alone. Moreover, the reach trajectory of the shared avatar was straighter than that of either participant and correlated with their subjective embodiment of the avatar. Finally, the jerk of the avatar's hand was less than either participant's own hand, both when they reached alone and in the shared body. Movement straightness and hand jerk are well known characteristics of human reach behavior, and our results suggest that during body sharing, humans prioritize these movement characteristics of the shared body over their own.

## Highlights

• We embodied two human participants within a shared avatar in virtual reality
• Movements of the shared avatar were the average of the participant's movements
• Avatar's hand movements were straighter and less jerky than participant's
• Humans prioritize movement of a shared body over their own

## Graphical Abstract

**Figure:**
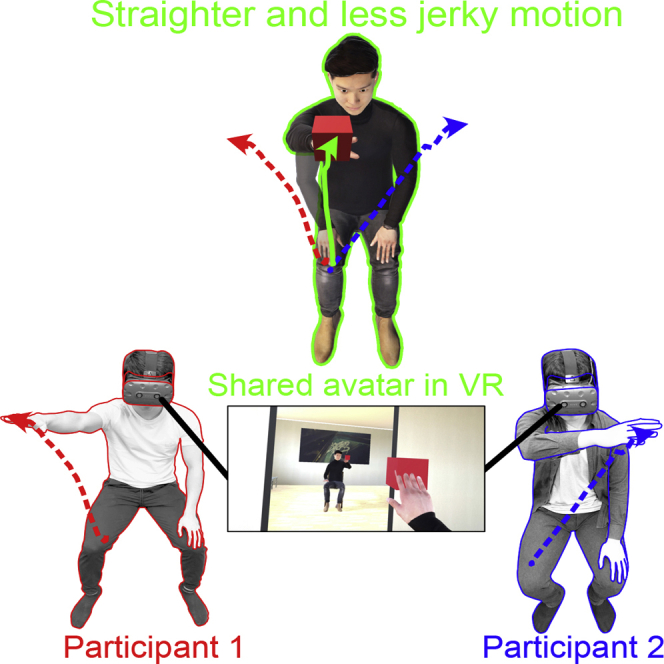

## Introduction

The use of the cyber space has seen a substantial expansion through the COVID-19 crisis. The cyber space enables us to work together at distant places and can even enable us to interact with remote environments and individuals by “embodying” virtual avatars, as well as real avatars, like robots. Such interactions using avatars is seen as a major future mode of communication between people. But for these to be possible, we need to understand the limits of avatar embodiment and how these embodiments affect the human users in terms of behaviors and emotions.

A plethora of studies have shown that, given the right sensory and/or motor stimulations, humans can elicit embodiment of virtual and physical objects other than their own body. Embodiment has been shown toward not only virtual bodies that are visually similar to one's own body (Gonzalez-Franco et al., 2010) but also toward bodies or objects that differ from one's own (Aymerich-Franch and Ganesh, 2016) in terms of size (Banakou et al., 2013; Tajadura-Jiménez et al., 2017; Falconer et al., 2014; Falconer et al., 2016), skin color (Peck et al., 2013), and arm length (Kilteni et al., 2012), as well as when the body is that of a robot (Aymerich-Franch et al., 2017; Aymerich-Franch, Petit, Ganesh and Kheddar, 2016a, 2016b; Aymerich-Franch et al., 2015; Aymerich-Franch, Petit, Ganesh and Kheddar, 2016a, 2016b). Furthermore, ownership can also be induced when different body parts are re-associated (Kondo et al., 2020) or even when the body is partially invisible (Kondo et al., 2018). Crucially, while these illusory bodies' appearances were different from the participants' own body, only few rare studies (like Burin et al., 2019; Kokkinara et al., 2016) have investigated the scenario where the movements of the illusory body did not correspond to the movement of the actual body. And even in these studies, the control of the body was still determined by a sole participant. Furthermore, in each of these studies participants perceived sole ownership of the body.

On the other hand, few studies have examined the case in which several people share a body (or avatar), or a limb, where the movement of an artificial body (or limb) is determined by more than one individual. Sharing thus enables multiple individuals to collaborate and contribute in the task performed by an avatar potentially decreasing the workload for each user and improving task performance. Sharing of body parts has been shown to lead to changes in the perceived ownership and agency (Fribourg et al., 2020; Hagiwara et al., 2019). On the other hand, it still remains unclear whether and how shared embodiment affects an individual's motor behavior. Here, we examined this issue using an arm reaching task in virtual reality, performed by a shared avatar who's arm movement was the average of arm movements by the two participants who share it.

**Figure 1:**
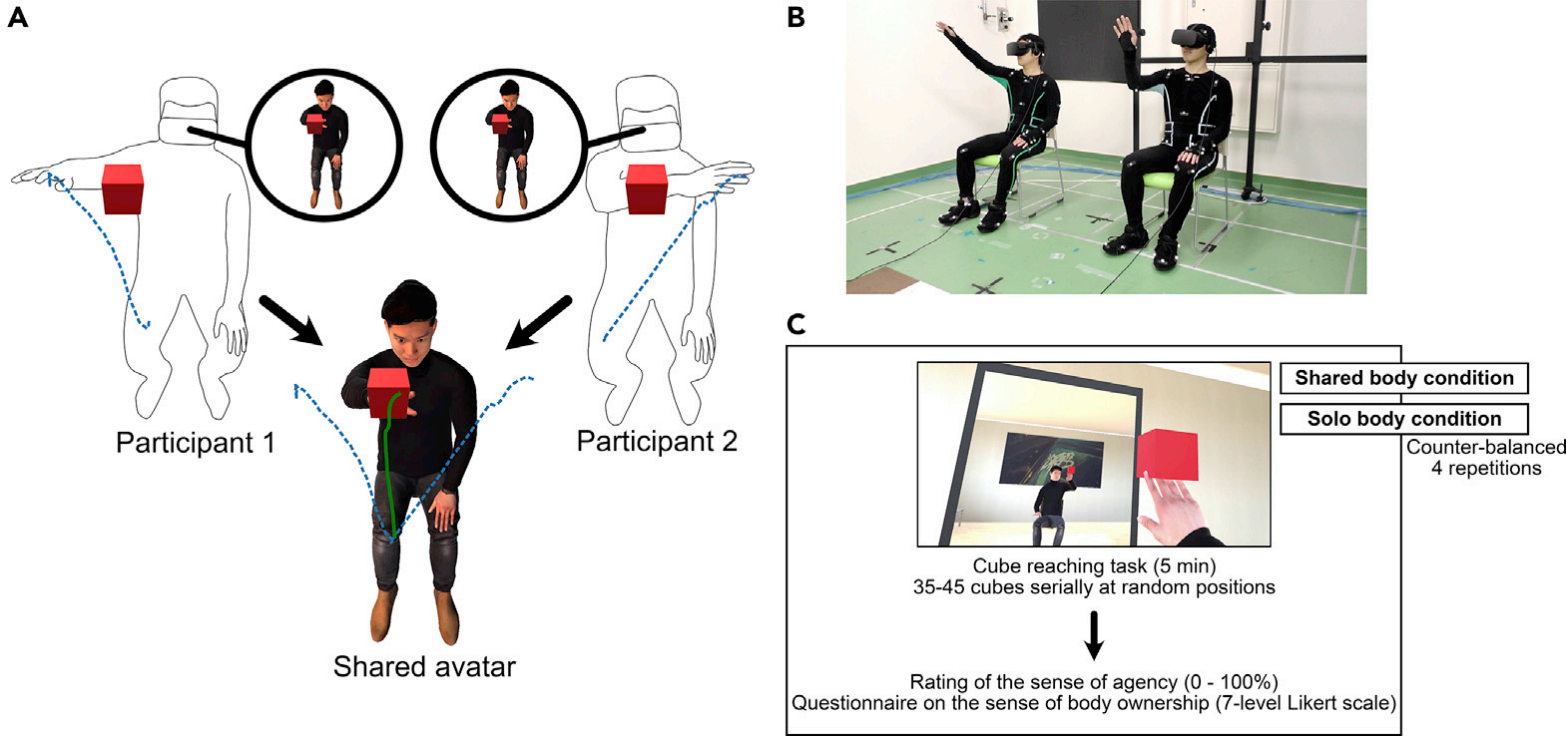
Shared Avatar Setup (A) The avatar's arm movement was developed by averaging the arm movements performed by the two participants in the Shared-body condition. Both participants observed the same shared avatar through their HMD. (B) The two participants wore the motion capture suites and HMD and performed the experiment. (C) Cube reaching tasks were performed for 5 min followed by the rating of the sense of agency and body ownership. The Shared-body condition and the Solo-body condition were repeated four times in counter-balanced order in the within-participant design.

Motor neuroscience studies have shown that individual human movement trajectories are a result of sensori-motor planning and control (Wolpert et al., 2011; Franklin and Wolpert, 2011; Ganesh and Burdet, 2013) as well as memory-related processes (Diedrichsen et al., 2010; Ganesh and Burdet, 2013; Ganesh et al., 2010). Considering these results, in this study we investigated what the individuals prioritize when they share a virtual body, specifically whether they still optimize their own movements or whether they optimize the movements of the avatar, and, consequently, how the sharing affects the avatar control. Here, we chose to investigate this issue during arm reaching, that is well-established empirical task to investigate motor behavior. Previous studies have shown that point-to-point arm reaching is roughly straight and tends to minimize the jerk measured at the hand (Flash and Hogan, 1985). Hence, here we compared the reach movement straightness and hand jerk exhibited by human individuals when they were embodied in individual avatars (Solo-body condition) with their hand movements when they were embodied in a same avatar (Shared-body condition), with the avatar movements representing their average hand movement (Figure 1, please also see Video S1). To anticipate our results, we observed that individual's movements change in the Shared-body condition (relative to the Solo-body condition) to minimize the trajectory length and jerk of the avatar hand, rather than that of their own hand.

Document S1. Trasnsparent Methods and Figures S1–S3

## Results

### Perceived Sense of Agency and Ownership to the Shared Body

Our experiment was performed in a virtual reality (VR) environment. The participants performed our experiment in dyads. They worked in two conditions. In each condition they were presented with a virtual avatar that replaced their own body in VR and were required to make reaching movements with their right hand, toward target cubes presented at various locations in front of both of them (see Transparent Methods for details). Both participants were presented with the same target at any time. In the *Solo-body* condition, the participant arm movement was replicated on the avatar, whereas in the *Shared-body* condition, the avatar's arm movement was the average of the arm movements performed by the two participants in the dyad (see Transparent Methods for details).

After each condition, the participants were asked to rate the sense of agency (0%–100%: 0 is “I did not control the avatar hand at all”; 100 is “I fully controlled the avatar hand”) and the sense of body ownership (Questionnaire: I felt as if the avatar's body I saw was my body; −3 to +3; 7 level Likert scale. −3 is “I did not feel it at all”; +3 is “I felt it extremely strongly”).

**Figure 2:**
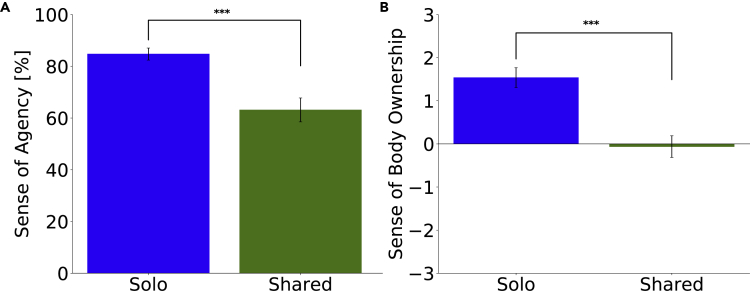
Sense of Agency and Body Ownership Perceived sense of agency was more than 50%, and the Solo-body condition was better than the Shared-body condition both in the sense of agency and the sense of body ownership. (A) Rating of the sense of agency. A Wilcoxon signed-rank test showed that the sense of agency was significantly higher in the Solo-body condition than in the Shared-body condition (∗∗∗p < .001). In the Solo-body condition, the sense of agency was significantly lower than 100%, and in the Shared-body condition, the sense of agency was significantly higher than 50%. The sense of agency was higher in the Solo-body condition than in the Shared-body condition. (B) Likert scale data of the sense of body ownership. A paired t test showed that the sense of body ownership was significantly higher in the Solo-body condition than in the Shared-body condition (∗∗∗p < .001). Bar plots in the figure show means +/− SE measures.

A Wilcoxon signed-rank test was conducted on the rated sense of agency to compare the Solo-body and Shared-body conditions because the data significantly deviated from normality (Shapiro-Wilk test, W = 0.828, p = .002). We found that the sense of agency was significantly higher in the Solo-body condition than in the Shared-body condition (W(19) = 190.000, p < .001, d = 1.000, Figure 2A). We conducted a Wilcoxon signed-rank test to test whether the rated sense of agency was different from the actual weights (100% in the Solo-body condition, 50% in the Shared-body condition). In the Solo-body condition, the sense of agency was significantly lower than 100% (W(19) = 0.000, p < .001, d = −1.000). Interestingly the sense of agency was significantly higher than 50% in the Shared-body condition (W(19) = 153.000, p = .021, d = 0.457), even though each participant contributed equally to the avatar movements.

A paired t test (one-sample t test) was conducted on the rated sense of body ownership in the Solo-body and Shared-body conditions because the data did not deviate from normality (Shapiro-Wilk test, W = 0.916, p = .083). As would be expected, a paired t test between the Solo-body and Shared-body conditions indicated that the sense of body ownership was significantly higher in the Solo-body condition than in the Shared-body condition (t(19) = 5.643, p < .001, d = 1.45, Figure 2B).

### Avatar Hand Movements Were Straighter Than That of Participants

We evaluated the straightness of the hand reach by measuring *hand reach deviation (D)*, which was defined as the difference between the length of the hand movement trajectory and the straight line joining the start and endpoint of the hand during a reach (See Transparent Methods and Figure S1 for detail). We evaluated the hand reach deviation by the participants in the Solo-body condition (D^solo^_human_), the actual hand reach deviation by the same participants in the Shared-body condition (D^shared^_human_) and the hand reach deviation by their shared avatar in the Shared-body condition (D^shared^_avatar_).

**Figure 3:**
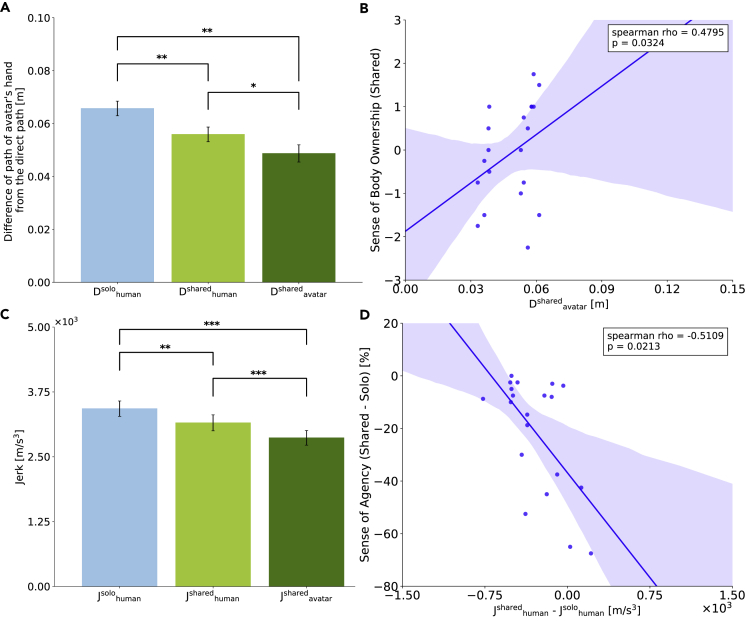
Avatar Hand Movements were Straighter and Exhibited Less Jerk (A) Difference of path of participant's hand (Solo-body condition, Shared-body condition) and shared body avatar's hand from the direct path. Holm's post hoc tests after a one-way repeated measures ANOVA were conducted (∗p < .05, ∗∗p < .01). D^solo^_human_ was significantly larger than D^shared^_human_, indicating that in the Shared-body condition the participants adopted a straighter path than when they performed in the reach alone. D^shared^_avatar_ was significantly smaller than both D^solo^_human_ and D^shared^_human_, indicating that the avatar attained hand trajectories that were straighter than the individual participant when they reached alone, as well as when they reached in the Shared-body condition. (B) Data are represented as each participant's D^shared^_avatar_ (horizontal axis) and the rated sense of body ownership in the Shared-body condition (vertical axis). The sense of body ownership in the Shared-body condition positively correlated with the hand reach deviation D in the Shared-body condition (please also see the rank order plot in Figure S2). (C) Average jerk in reaching. Tukey's post hoc tests with Kenward-Roger degrees of freedom approximation after a one-way repeated measures ANOVA with ART were conducted (∗∗p < .01, ∗∗∗p < .001). J^shared^_avatar_ was smaller than both J^solo^_human_ and J^shared^_human_. Also, J^shared^_human_ was significantly smaller than J^solo^_human_. Thus, the shared avatar's movements were smoother than when they were solo. (D) Data are represented as each participant's difference between J^shared^_human_ and J^solo^_human_ (horizontal axis) and difference of the rated sense of agency between the Shared-body condition and the Solo-body condition (vertical axis). The change in the sense of agency between the Shared-body and Solo-body conditions inversely correlated with the change in hand jerk between the conditions (please also see the rank order plot in Figure S3). Bar plots in (A) and (C) show means +/− SE measures.

A one-way repeated measures ANOVA across the three reach deviations (D^solo^_human_, D^shared^_human_, D^shared^_avatar_) was conducted because the data did not deviate from normality (Shapiro-Wilk test, D^solo^_human_: W = 0.932, p = .468, D^shared^_human_: W = 0.928, p = .433, D^shared^_avatar_: W = 0.849, p = .056) or sphericity (Mauchly's sphericity test, p = .319). We found a significant main effect (F(2, 18) = 19.278, p < .001, $\eta_{p}^{2}$ = 0.682, Figure 3A). Holm's post hoc test indicated that D^solo^_human_ was significantly larger than D^shared^_human_ (t(9) = 4.150, adj.p = .005, d = 1.312), indicating that, in the Shared-body condition, the participants adopted a straighter path than when they performed in the reach alone. Interestingly, D^shared^_avatar_ was smaller than both D^solo^_human_ (t(9) = 5.054, adj.p = .002, d = 1.598) and D^shared^_human_ (t(9) = 2.998, adj.p = .015, d = 0.948) indicating that the avatar attained hand trajectories that were straighter than the individual participant when they reached alone, as well as when they reached in the Shared-body condition.

### Avatar Hand Jerk Was Less Than That of Individual Participants

Next, we calculated the average hand jerk in reaching in the same three cases (J^solo^_human_, J^shared^_human_, J^shared^_avatar_; Figure 3C). A one-way repeated measures ANOVA with ART (aligned rank transformation) procedure (Wobbrock et al., 2011) was conducted because the data significantly deviated from normality (J^shared^_avatar_: W = 0.823, p = .028). We found a significant main effect (F(2, 18) = 37.238, p < .001, $\eta_{p}^{2}$ = 0.805) across the three measures. Tukey's post hoc tests with Kenward-Roger degrees of freedom approximation (Kenward and Roger, 1997) indicated that J^shared^_avatar_ was significantly smaller than both J^solo^_human_ (t(18) = 8.629, adj.p < .001, d = −3.86) and J^shared^_human_ (t(18) = 4.438, adj.p = .001, d = −1.98). Also, J^shared^_human_ was smaller than J^solo^_human_ (t(18) = 4.191, adj.p = .002, d = −1.87). These results show that the shared avatar's movements were smoother that when they were solo. Furthermore, in the Shared-body condition, the participants might achieve smoother movements with the avatar than with their own hands.

### Task Performance: Reaction of Avatar Was Better, Target Error Was Similar

**Figure 4:**
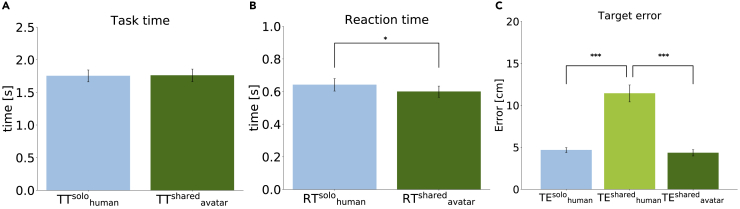
Performance by Avatar Changed Compared with Individual Participants (A) Participants' task time in the Solo-body condition (TT^solo^_human_) and the shared avatars' task time in the Shared-body condition (TT^shared^_avatar_). There was no difference. (B) A paired t test showed that the shared avatar's reaction time in the Shared-body condition (RT^shared^_avatar_) was significantly faster than the participants' reaction time in the Solo-body condition (RT^solo^_human_) (∗p < .05). (C) Tukey's post hoc tests with Kenward-Roger degrees of freedom approximation after a one-way repeated measures ANOVA with ART showed that the target errors of hand reaching TE^shared^_avatar_ and TE^solo^_human_ were significantly smaller than TE^shared^_human_ (∗∗∗p < .001). TE^shared^_avatar_ was slightly smaller than TE^solo^_human_ but the difference did not reach significance. Bar plots in the figure show means +/− SE measures.

We quantified the task performance in our experiment by evaluating the *task time*, *reaction time*, and *target error*. We compared the participants' task time in the Solo-body condition (TT^solo^_human_) and the shared avatars' task time in the Shared-body condition (TT^shared^_avatar_, Figure 4A). The task time was defined as a time between the appearance of the target and the hand's touching the target. A paired t test was conducted on the task time because the data did not deviate from normality (Shapiro-Wilk test, W = 0.950, p = .667). There was no difference (t(9) = 0.174, p = .866, d = 0.055). We similarly compared the participants' reaction time in the Solo-body condition (RT^solo^_human_) and the shared avatars' reaction time in the Shared-body condition (RT^shared^_avatar_, Figure 4B). The reaction time was defined as the time between the appearance of the target and the time at which the hand velocity goes over 10% of the maximum velocity. A paired t test was conducted on the reaction time because the data did not deviate from normality (Shapiro-Wilk test, W = 0.942, p = .574). The shared avatars' reaction time was shorter than the participants' reaction time (t(9) = 2.357, p = .043, d = 0.745).

Target error was defined as the difference between the endpoint of the participant's reach and the center of the target object and was again calculated for the three cases as before (TE^solo^_human_, TE^shared^_human_, and TE^shared^_avatar_; Figure 4C). A one-way repeated measures ANOVA with ART procedure was conducted because the data significantly deviated from normality (TE^shared^_human_: W = 0.774, p = .007). We found a significant main effect (F(2, 18) = 117.839, p < .001, $\eta_{p}^{2}$ = 0.929) in the target error across the cases. Tukey's post hoc tests with Kenward-Roger degrees of freedom approximation indicated that TE^shared^_avatar_ was significantly smaller than TE^shared^_human_ (t(18) = 14.017, adj.p < .001, d = −6.27). TE^solo^_human_ was significantly smaller than TE^shared^_human_ (t(18) = 12.430, adj.p < .001, d = 5.56). TE^shared^_avatar_ was slightly smaller than TE^solo^_human_, but the difference did not reach significance (t(18) = 1.587, adj.p = .277, d = −0.71). These results show that the participant's reach performance improved when sharing the avatar body, in terms of the reaction time, while remaining same in terms of the target error. In the Shared-body condition, the avatar task performance improved, even when individual participant errors increased.

### Interpersonal Distance Changes: An Argument against Averaging Effects

In our experiment we observed that the participants attained a straighter (Figure 3A) and smoother hand trajectories (Figure 3C) than they could do with their own hands under the Solo-body condition. They could also achieve similar target errors with the avatar, as the Solo-body condition (Figure 4C). It is, however, important to note that the participant movements suffer from sensory as well as motor noise (Harris and Wolpert, 1998), and the avatar movement is generated by averaging the hand movements of the participants. Therefore, there is the possibility that, in fact, the participants behave the same in the Solo-body (IP^solo^_human_) and Shared-body (IP^shared^_human_) conditions and that the improvements in straightness, jerk, and target errors we observed are just a consequence of the averaging of the individual participant movements.

**Figure 5:**
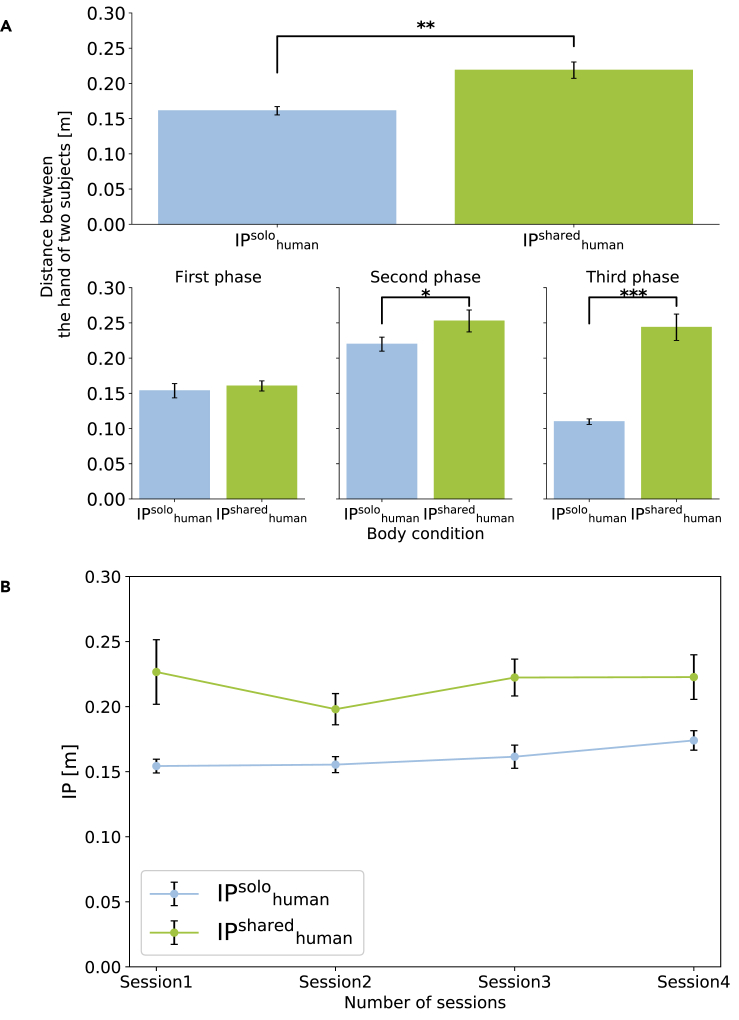
Interpersonal Distance was Larger in the Shared-Body Condition Than in the Solo-Body Condition (A) Distance between the hand of two participants (top row: Full phase, bottom row: First phase, Second phase, Third phase). IP^solo^_human_ was significantly lower than IP^shared^_human_, indicating that, although participants took similar hand trajectories in the Solo-body condition, their hand trajectories deviated away from one another in the Shared-body condition such that the inter-participant distance was higher. (Full phase) A Wilcoxon signed-rank test showed that IP^shared^_human_ was significantly larger than IP^solo^_human_ (∗∗p < .01). (First phase) A paired t test did not show significant difference. (Second phase, Third phase) Paired t-tests showed that IP^shared^_human_ was significantly larger than IP^solo^_human_ (∗p < .05, ∗∗∗p < .001). (B) There was a significant main effect between the IPs in the Solo-body condition and the Shared-body condition but not main effect of sessions (hence trials) or an interaction (a two-way repeated measures ANOVA with ART procedure). Bar plots in the figure show means +/− SE measures.

To ensure that the participant behaviors did change between the Shared-body and Solo-body, we evaluated the inter-participant distance (IP) between the participants' hand trajectories during reaching in the Shared-body and Solo-body conditions (Figure 5A). A Wilcoxon signed-rank test was conducted because the data significantly deviated from normality (Shapiro-Wilk test, W = 0.826, p = .030). We found that IP^solo^_human_ was significantly lower than IP^shared^_human_ (W(9) = 0.000, p = .002, d = 1.000). This result indicates that, although participants took similar hand trajectories in the Solo-body condition (even though they embodied separate avatars), their hand trajectories deviated away from one another in the Shared-body condition such that the inter-participant distance was higher. We also compared the evolution of IP^solo^_human_ and IP^shared^_human_ over three phases of the reach (Figure 5A). These three phases were obtained by dividing the reaching between the appearance of the target and the hand's touching the target into three equal parts in time. Because the data did not deviate from normality (Shapiro-Wilk's normality test, phase 1: W = 0.990, p = .996; phase 2: W = 0.921, p = .365; phase 3: W = 0.851, p = .060), we conducted paired (one-sample) t-tests. IP^solo^_human_ and IP^shared^_human_ were similar during the first phase of the participant reach (t(9) = 1.064, p = .315, d = 0.336), but showed considerable difference in the second and third phase of the movement, near the targets (t(9) = 3.155, p = .012, d = 0.998; t(9) = 7.037, p < .001, d = 2.225).

We also compared the modifications in IP over trials (Figure 5B). Participants worked in Solo-body condition and Shared-body condition across eight sessions (each condition presented four times). We performed a two-way repeated measures ANOVA across these sessions with ART procedure because the data significantly deviated from normality (Shapiro-Wilk test, Session 1 of IP^shared^_human_: W = 0.805, p = .016, Session 2 of IP^shared^_human_: W = 0.833, p = .037). A significant main effect (F(1, 9) = 55.216, p < .001, $\eta_{p}^{2}$ = 0.772) between the IPs in the Solo-body condition and the Shared-body condition was found. There was no main effect of sessions (hence trials) (F(3, 27) = 2.088, p = .111, $\eta_{p}^{2}$ = 0.179) and no interaction (F(3, 27) = 0.578, p = .632, $\eta_{p}^{2}$ = 0.095).

These results clearly show that the participant behaviors changed significantly between the Solo-body and Shared-body conditions.

Overall, the results in Figures 3 and 4 suggest that the increase in straightness (Figure 3A), decrease in jerk (Figure 3C), and similar target error (Figure 4C) by the avatar compared with the individuals in the Solo-body condition cannot be explained by the averaging of trajectories alone. On the other hand, these results suggest that the participants changed their behavior in the Shared-body condition to optimize these movement variables of the avatar, while ignoring the same on their own hands.

## Discussion

To evaluate how the control of a shared avatar affects the motor behavior of the controlling participants, we developed an avatar reaching task in virtual reality. We compared the participant hand trajectories in the Solo-body condition, in which the avatar was controlled alone by individuals, with the Shared-body condition in which the avatar movement was the average of the movements made by two participants. Movement straightness and jerk are known to be optimized by individuals for their hand movements (Flash and Hogan, 1985). However, interestingly, here we observed that the shared avatar hand trajectories were straighter (Figure 3A) and displayed less jerk (Figure 3C) than the hand movements by the participants, both alone and when they were controlling the avatar. The shared avatar also displayed faster reaction times than participants alone (Figure 4B). These results indicate that participants prioritize the optimization of the hand trajectories by the shared avatar over their own individual arm trajectories.

A previous study that examined reaching with a dynamic tool showed that humans optimize the movement of the endpoint of the tool rather than their hand (Dingwell et al., 2002). Although our results here look seemingly similar, there are two fundamental differences. Primarily, the previous task involved a single person controlling and working with the tool. In contrast, the current task examined shared control, where two individuals cooperated to achieve a common task by the avatar they controlled. Second, we observed that the reach task performance was significantly improved while using the shared avatar (Figure 4B). This behavior was not observed during the tool learning task. These differences in fact suggest the behavior in the Shared-body condition to be similar to that observed during human inter-personnel interactions, where low-impedance interactions have been shown to benefit task performance (Ganesh et al., 2014; Takagi et al., 2017; Takagi et al., 2018). Inter-personal benefits are, however, believed to be enabled by the prediction of the partner's behavior utilizing the haptic cues (Takagi, et al., 2017). Although in our task there obviously was no haptic interaction, we believe partner prediction as the reason for the straighter path of the avatar. The avatar movement in out setup was the consequence of movements made by the two dyad participants. This forced the dyad participants to cooperate, and develop an avatar trajectory together, in order to perform reaches successfully. However, this demands that the individuals predict each other's preferred avatar trajectory. We believe that the choice of the straight avatar trajectory was driven by the fact that this choice optimizes the predictability of one's partner's avatar trajectory choice. Subsequently, the lower jerk and endpoint error of the avatar reaches are probably the consequence of the straighter trajectory. Further studies are, however, required to prove this hypothesis.

Body ownership decreases when there is a mismatch between visual and motor information (Sanchez-Vives et al., 2010). This explains why in the Shared-body condition, where the avatar movement is a consequence of movements by two partners, the sense of body ownership is less than in the Solo-body condition. Interestingly, we found that the reported level of ownership in the Shared-body condition positively correlated with the hand reach deviation D in the Shared-body condition (Spearman's rank-order correlation ρ = 0.4795, p = .032; Figure 3B), and the change in the reported level of agency between the Shared-body and Solo-body conditions inversely correlates with the change in hand jerk between the conditions (Spearman's rank-order correlation ρ = −0.5109, p = .023; Figure 3D). In these results, we adopted Spearman's rank-order correlation because the data significantly deviated from normality (Shapiro-Wilk test, D^shared^_avatar_: W = 0.828, p = .002; Sense of agency (Shared-Solo): W = 0.828, p = .002). Although a previous study has shown the observation of an embodied limb can implicitly disturb the movements of a person (Burin et al., 2019), our results are probably the first to suggest that participants optimize the movements of the embodied limb rather than their own, which is probably also a cause of the observations in Burin et al. (2019). Pyasik, Furlanetto, and Pia (2019) propose the idea that only seeing the own body moving would be enough to activate the neurocognitive processes sub-serving action preparation based on the findings that the body ownership affects the sense of agency (e.g., Banakou and Slater, 2014; Kokkinara et al., 2016; Burin et al., 2019). Our results show that the low-level movement control can be systematically affected by the level of embodiment felt toward a limb. Accordingly, it might be supposed that seeing the shared body (or sense of body ownership to the shared body) can directly affect the motor control process as well as the sense of agency. A better understanding of the relation between embodiment and motor control promises new application of the shared body concept as a virtual avatar, like in our study, or as a collaborating robot.

### Limitations of the Study

Although we find that the hand trajectories of the shared avatar are both straighter and less jerky than the solo hand trajectories, our results cannot clarify the causality, that is, whether the participant's goal is to have a straighter avatar trajectory, which subsequently results in less jerky hand trajectory, or, conversely, they aim for less jerk, which results in a straighter trajectory. Further studies are required to clarify this issue. However, in either case, the key goal of this work was to show that the participants are able to make straighter trajectories and better minimize the hand jerk of the shared avatar than their own.

### Resource Availability

#### Lead Contact

Further information and requests for resources should be directed to and will be fulfilled by the Lead Contact, Takayoshi Hagiwara (takayoshi.hagiwara.18@gmail.com).

#### Materials Availability

This study did not generate new unique reagents.

#### Data and Code Availability

Original data for figures in the paper is available at Mendeley Data https://doi.org/10.17632/w9n6s2kcmc.1.

## Methods

All methods can be found in the accompanying Transparent Methods supplemental file.

## Supplemental Information

Video S1. Related to Figure 1, Methods of the Experiment The video explains the methods including apparatus, stimuli, and procedure.
