# Supplementary material for: Individuals Prioritize the Reach Straightness and Hand Jerk of a Shared Avatar over Their Own
Source: iScience. 2020 Nov 10;23(12):101732. doi: 10.1016/j.isci.2020.101732 (PMC7756142; doi:10.1016/j.isci.2020.101732)
Supplement: Document S1. Trasnsparent Methods and Figures S1–S3 [file mmc1.pdf]

iScience, Volume *23*

Supplemental Information

Individuals Prioritize the Reach Straightness and Hand Jerk of a Shared Avatar over Their Own

Takayoshi Hagiwara, Gowrishankar Ganesh, Maki Sugimoto, Masahiko Inami, and Michiteru Kitazaki

Supplemental Figures and Legends

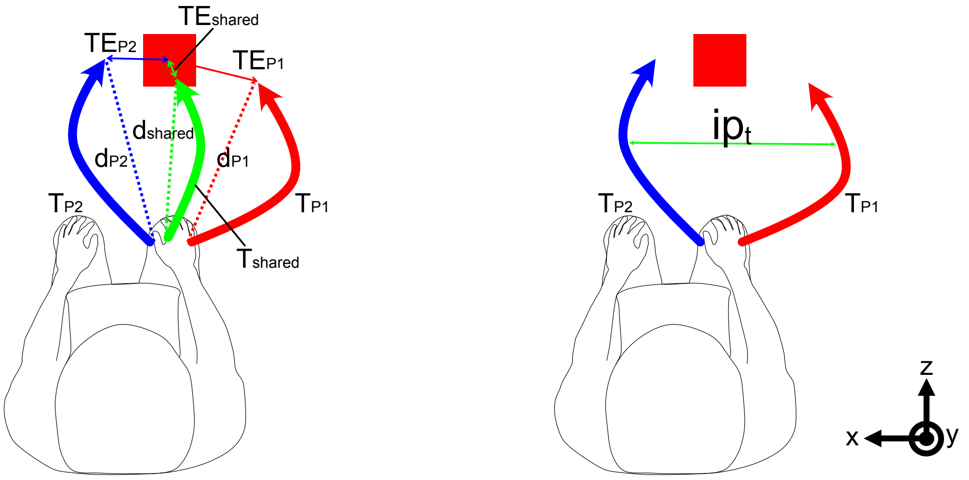

Figure S1: Measures for reaching analysis. Related to the hand reach deviation (D), the target error (TE) and interpersonal distance (IP). Related to Figure 3A, Figure 4C, Figure 5.

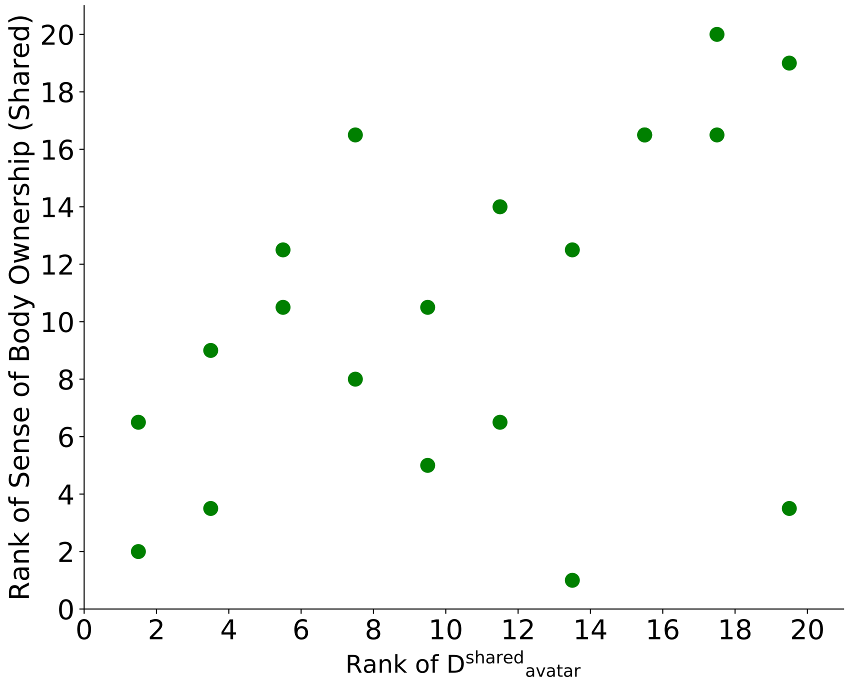

Figure S2: Data are represented as each participant's rank order in ascending order for D^{shared}_{avatar} and the sense of body ownership in the Shared-body condition. Related to Figure 3B.

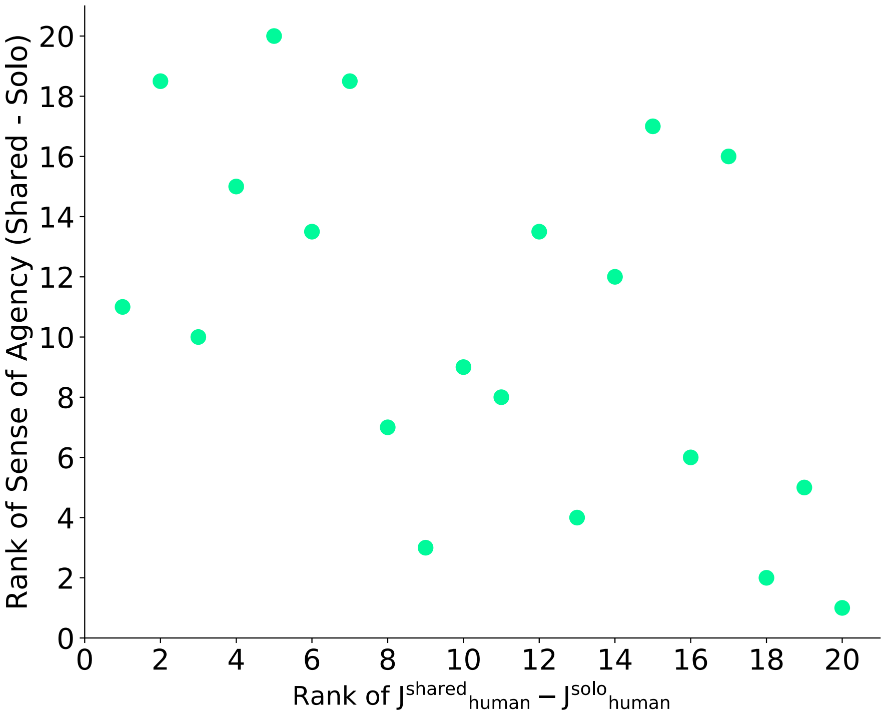

Figure S3: Data are represented as each participant's rank order in ascending order for the change in hand jerk between the Shared-body and Solo-body conditions and the change in the sense of agency between the conditions. Related to Figure 3D.

Transparent Methods

Participants

Twenty volunteers participated in the experiment (all male, mean 21.35 years old ± 1.5 standard deviation (SD)). The sample size was based on previous embodiment (Fribourg et al., 2020) and motor control (Ganesh, et al., 2014) studies involving interaction between human dyads. This number corresponds to an effect size of 0.7, alpha = 0.05, power = 0.8 using the G*Power 3.1 (Faul, Erdfelder, Lang, & Buchner, 2007; Faul, Erdfelder, Buchner, & Lang, 2009). All participants have normal binocular vision and physical ability. They gave written informed consent before the experiment. All the experiments were approved by the Ethical Committee for Human-Subject Research at Toyohashi University of Technology and were performed in accordance with this committee's guidelines and regulations.

Setup and Apparatus

The participants took part in the experiment as dyads. They sat in chairs with their backs straight, and with their hands resting on their knees (initial posture). The movements of two participants were measured by a motion-capture system (Vicon Bonita10, 12 cameras, 1024 x 1024 pixels, 250 fps, focal length: 4-12 mm, F/1.4-CLOSE, angle of view: 26.41 x 26.41 deg), and processed in a computer (HP Z440 Workstation, OS: Windows7, CPU: Intel(R) Xeon E5-1620 v3, 3.5GHz, RAM: 32GB, GPU: NVIDIA Quadro 5000 (2560MB GDDR5)) with middle-level software (Vicon Blade 3.4.1, Vicon Pegasus 1.2.2). Two computers (PC1: DELL XPS 8930, OS: Windows10, CPU: Intel(R) Core i7-8700 3.2GHz, RAM: 16GB, GPU: NVIDIA GeForce GTX 1080 (8192MB GDDR5X), PC2: DELL Alienware Aurora, OS: Windows10, CPU: Intel(R) Core i5-6400 2.7GHz, RAM: 16GB DDR4 2667MHz, GPU: NVIDIA GeForce GTX 1080 (8192MB GDDR5X)) received the processed information of the motion capture data, and presented the virtual environment on head-mounted displays (HMD: Oculus Rift CV1, 1080 x 1200 pixels, 90 x 110 deg, 90 Hz refresh) of both participants. The virtual environment (VE) was created using Unity (2017.3.0f3).

Stimuli and conditions

A virtual three-dimensional (3D) space and a male avatar were rendered using Unity

and presented on the HMD. The height of the male avatar was 175 cm and the length of the right arm was 65.8 cm. The avatar sat on a chair in a room (4.5 m depth x 5.5 m wide x 2.5 m high), and the window was placed on the left side as seen from the avatar. A mirror (1.4 m wide x 2.2 m high) was presented in front of the avatar in the VE so that the participants could see their movement and the target object.

The movement of the avatar was a weighted sum of two participant's motions. The position and rotation of the bones of each part of the body were received from the motion capture system, and applied to the corresponding bones of the avatar. The weights reflected in the avatar was set so that the sum of the two participants was 100%. The participants looked at the avatar in the first-person perspective.

The target object was a red cube (10 x 10 x 10 cm). A burst sound was presented when the first joint of the avatar's middle finger touched the center of the object. The cube disappeared immediately after the touch, then a new cube appeared in a random position 5 s later. Participants were instructed to touch the target object while actually looking at it, rather than looking at the target object in the mirror. The target object appeared at a horizontal angle of ± 70 deg and a vertical angle of ± 45 deg from the shoulder position of the shared avatar. It appeared within a radius of 65 cm from the shoulder but did not appear less than 35 cm to the participant.

For each condition, a dyad performed the cube reaching task for 5 min, and the participants were asked to reach the cube with their right hand. The weights utilized in each condition were as follows- For the Solo-body condition: Participant 1: 100%, Participant 2: 100%, and for the Shared-body condition: Participant 1: 50%, Participant 2: 50%. The participants were not aware of the weights, and the condition order was counter-balanced with 4 repetitions. In the Solo-body condition, two participants each saw an avatar that represented their own movements.

The movements of the two participants were recorded by the motion capture system, and transferred to Unity programs in two computers. The motion data were under-sampled at 90 Hz (same timing as display refresh), and the positions of a specific parts of the participants and the avatar (head, neck, three parts in spine (divided in equal length), left shoulder, left upper arm, left forearm, left hand, right shoulder, right upper arm, right forearm, right hand, and three parts in middle finger of right hand (divided in equal

length) were extracted. We used only the positions of tip of the middle finger of the right hand.

Procedure

The participants were seated in chairs with their backs straightened, and their hands were put on their knees (initial posture). For each trial, a dyad performed the cube reaching task for 5 min, and the participants were asked to reach the cube with the right hand. After the task, the participants were asked to rate the sense of agency (Questionnaire: How much did you feel as if you controlled the avatar hand? Please give a ratio of the sense of agency between 0 and 100%. 0 is "I did not control the avatar hand at all", 100 is "I fully controlled the avatar hand"). Then, they were asked to rate the sense of body ownership (Questionnaire: I felt as if the avatar's body I saw was my body; -3 to +3; 7 level Likert scale. -3 is "I did not feel it at all", +3 is "I felt it extremely strongly"). These items were presented on a computer screen, and participants answered the sense of agency with a keyboard (0-100), and answered the sense of body ownership by choosing from the pull-down menu (-3 to +3). Two conditions (Shared-body condition and Solo-body condition) were repeated 4 times in counter-balanced order.

Measurements for reaching analysis

The participants (P1, P2) performed the experiment in dyads (see Supplementary Figure 1). They worked in two conditions. In the Solo-body condition, the participant arm movement was replicated on the avatar, while in the Shared-body condition, the avatar's arm movement was the average of the arm movements performed by the two participants in the dyad.

$\mathrm{T_{P1}}$, $\mathrm{T_{P2}}$ and $\mathrm{T_{shared}}$ indicate the length of trajectory (T) of the participant P1, the participant P2, and the shared avatar (shared), respectively. t indicates the indices of the data frame collected at 90 Hz. F indicates the number of all frames in a reaching. The initial point is defined as the hand position when the target cube appears. The end point of the reach is defined as the point at which the hand velocity falls below 10% of the maximum velocity for the first time. T is calculated as follows:

$$\mathrm{T} = \sum_{t=0}^{\mathrm{F}} \sqrt{(x_{t+1} - x_t)^2 + (y_{t+1} - y_t)^2 + (z_{t+1} - z_t)^2}$$

$\mathrm{d_{P1}}$, $\mathrm{d_{P2}}$ and $\mathrm{d_{shared}}$ indicate the direct distance of the participant P1, the participant P2, and the shared avatar, respectively from the initial hand point (x_0, y_0, z_0) to the end point $(x_{\mathrm{end}}, y_{\mathrm{end}}, z_{\mathrm{end}})$ during reach. d is calculated as follows:

$$\mathrm{d} = \sqrt{(x_{\mathrm{end}} - x_0)^2 + (y_{\mathrm{end}} - y_0)^2 + (z_{\mathrm{end}} - z_0)^2}$$

Then, we calculated the difference (D) between the length of the hand movement trajectory (T) and the direct distance (d).

$\mathrm{D^{solo}}_{\mathrm{human1}}$, $\mathrm{D^{solo}}_{\mathrm{human2}}$ and $\mathrm{D^{shared}}_{\mathrm{avatar}}$ indicate the difference between the length of the hand movement trajectory ($\mathrm{T_{P1}}$, $\mathrm{T_{P2}}$, $\mathrm{T_{shared}}$) and the direct distance ($\mathrm{d_{P1}}$, $\mathrm{d_{P2}}$, $\mathrm{d_{shared}}$). $\mathrm{D^{solo}}_{\mathrm{human1}}$ and $\mathrm{D^{solo}}_{\mathrm{human2}}$ indicate the hand reach deviation by the participants (P1, P2) in the Solo-body condition. $\mathrm{D^{shared}}_{\mathrm{avatar}}$ indicate the hand reach deviation by the shared avatar in the Shared-body condition.

$$\mathrm{D^{solo}}_{\mathrm{human1}} = \mathrm{T_{P1}} - \mathrm{d_{P1}}$$
$$\mathrm{D^{solo}}_{\mathrm{human2}} = \mathrm{T_{P2}} - \mathrm{d_{P2}}$$
$$\mathrm{D^{shared}}_{\mathrm{avatar}} = \mathrm{T_{shared}} - \mathrm{d_{shared}}$$

Target error (TE) was calculated as a performance measure.

$\mathrm{TE_{P1}}$, $\mathrm{TE_{P2}}$ and $\mathrm{TE_{shared}}$ indicate the difference between the end point of the participant's reach $(x_{\mathrm{end}}, y_{\mathrm{end}}, z_{\mathrm{end}})$ and the center of the target object (x_c, y_c, z_c). $\mathrm{TE_{P1}}$ and $\mathrm{TE_{P2}}$ indicate the target error (TE) of the participant P1 and P2, respectively. $\mathrm{TE_{shared}}$ indicate the target error (TE) of the shared avatar in the Shared-body condition. TE is calculated as follows:

$$\mathrm{TE} = \sqrt{(x_c - x_{\mathrm{end}})^2 + (y_c - y_{\mathrm{end}})^2 + (z_c - z_{\mathrm{end}})^2}$$

ip_t indicates the distance between the two participant's hand position at a frame

(Participant1: $x_{\mathrm{P}1t}, y_{\mathrm{P}1t}, z_{\mathrm{P}1t}$; Participant2: $x_{\mathrm{P}2t}, y_{\mathrm{P}2t}, z_{\mathrm{P}2t}$). ip is calculated as follows:

$$\mathrm{ip}_t = \sqrt{(x_{\mathrm{P}1t} - x_{\mathrm{P}2t})^2 + (y_{\mathrm{P}1t} - y_{\mathrm{P}2t})^2 + (z_{\mathrm{P}1t} - z_{\mathrm{P}2t})^2}$$

$\mathrm{IP}^{\mathrm{solo}}{}_{\mathrm{human}}$ indicate the average inter-participant distance by the participants in the Solo-body condition. $\mathrm{IP}^{\mathrm{shared}}{}_{\mathrm{human}}$ indicate the average inter-participant distance by the same participants in the Shared-body condition. IP is calculated as follows:

$$\mathrm{IP} = \frac{1}{\mathrm{F}}\sum_{t=0}^{\mathrm{F}} \mathrm{ip}_t$$

We calculated jerk of participant's and avatar's hand reaching.

$\mathrm{J}^{\mathrm{solo}}{}_{\mathrm{human}}$, $\mathrm{J}^{\mathrm{shared}}{}_{\mathrm{human}}$ and $\mathrm{J}^{\mathrm{shared}}{}_{\mathrm{avatar}}$ indicates the square root of the third order differential of the position. $\mathrm{J}^{\mathrm{solo}}{}_{\mathrm{human}}$ indicate the jerk by the participants in the Solo-body condition. $\mathrm{J}^{\mathrm{shared}}{}_{\mathrm{human}}$ indicate the jerk by the participants in the Shared-body condition. $\mathrm{J}^{\mathrm{shared}}{}_{\mathrm{avatar}}$ indicate the jerk by the shared avatar in the Shared-body condition. J is calculated as follows:

$$\mathrm{J} = \frac{1}{\mathrm{F}}\sum_{t=0}^{\mathrm{F}} \sqrt{\dddot{x}_t^{\,2} + \dddot{y}_t^{\,2} + \dddot{z}_t^{\,2}}$$

We calculated the task time (TT) and the reaction time (RT) for reaching time measures. The task time was defined as a time between the appearance of the target and the hand's touching the target. $\mathrm{TT}^{\mathrm{solo}}{}_{\mathrm{human}}$ indicates the participant' task time in the Solo-body condition, and $\mathrm{TT}^{\mathrm{shared}}{}_{\mathrm{avatar}}$ indicates the shared avatars' task time in the Shared-body condition. The reaction time was defined as the time between the appearance of the target and the time at which the hand velocity goes over 10% of the maximum velocity. $\mathrm{RT}^{\mathrm{solo}}{}_{\mathrm{human}}$ indicates the participants' reaction time in the Solo-body condition, and $\mathrm{RT}^{\mathrm{shared}}{}_{\mathrm{avatar}}$ indicates the shared avatar's reaction time in the Shared-body condition.

Analysis and statistics

All analyses were performed in RStudio and R software (Version 4.0.2, The R Project for Statistical Computing, https://www.r-project.org/) and JASP (Version 0.13.1, https://jasp-stats.org/). Aligned rank transformation (ART) procedure was performed with the "ARTool" package (https://depts.washington.edu/acelab/proj/art/index.html). All data groups were first tested for normality using the Shapiro-Wilk test before analysis. Following this, paired t-tests or repeated measures ANOVAs were utilized for the analysis of data that did not violate the assumption of normality (Shapiro-Wilk test, $p > .05$). Wilcoxon's signed-rank tests or repeated measures ANOVAs with ART were used for data that significantly deviated from the normality (Shapiro-Wilk test, $p < .05$). For correlation analysis, Spearman's rank-order correlations were used when either of both of the compared data groups significantly deviated from normality (Shapiro-Wilk test, $p < .05$).
